# Professional oral care in end-of-life patients with advanced cancers in a hospice ward: improvement of oral conditions

**DOI:** 10.1186/s12904-020-00684-0

**Published:** 2020-11-27

**Authors:** Ting-Ying Wu, Hsiu-Yueh Liu, Chien-Yi Wu, Hung-Cheng Chen, Shun-Te Huang, Ping-Ho Chen

**Affiliations:** 1 School of Dentistry, College of Dental Medicine, Kaohsiung Medical University, Kaohsiung, 80708 Taiwan; 2 Department of Oral Hygiene, College of Dental Medicine, Kaohsiung Medical University, Kaohsiung, 80708 Taiwan; 3 Department of Family Medicine, Kaohsiung Medical University Hospital, Kaohsiung Medical University, Kaohsiung, 80708 Taiwan; 4 Department of Nursing, Kaohsiung Medical University Hospital, Kaohsiung Medical University, Kaohsiung, 80708 Taiwan; 5 Division of Special Care Dentistry, Department of Dentistry, Kaohsiung Medical University Hospital, Kaohsiung, 80708 Taiwan; 6 Center for Cancer Research, Kaohsiung Medical University, Kaohsiung, 80708 Taiwan; 7 Cancer Center, Kaohsiung Medical University Hospital, Kaohsiung Medical University, Kaohsiung, 80708 Taiwan; 8 Institute of Biomedical Sciences, National Sun Yat-sen University, Kaohsiung, 80424 Taiwan

**Keywords:** End-of-life, Advanced cancers, Prospective intervention study, Professional oral care, Oral health assessment tool, Mucositis, Oral dryness, Oral debris

## Abstract

**Background:**

In end-of-life patients with advanced cancers, oral examination, oral care, and oral re-examination are crucial. Although oral symptoms are among the major complaints of end-of-life patients, few studies have focused on oral care in these patients. In this study, the association between oral symptoms and oral dryness among end-of-life patients was examined, and improvement of oral conditions after oral care interventions by a professional dentist was quantified.

**Methods:**

This prospective intervention study included 27 terminally ill patients with advanced cancers in a hospice ward. Professional oral care was administered every morning, and the improvement of oral conditions was assessed by comparing oral conditions before and after the intervention. Oral assessment was performed using the Oral Health Assessment Tool (OHAT) and Oral Assessment Guide. Oral dryness was evaluated through Clinical Diagnosis Classification of oral dryness and an oral moisture device. Oral cleanliness was evaluated using a bacterial counter, and tongue smears were collected for *Candida* examination; furthermore, oral function was recorded.

**Results:**

The presence of oral mucositis was closely associated with severe oral dryness (odds ratio [OR] = 14.93; 95% confidence interval [CI]: 1.95–114.38). The level of oral debris retention was significantly related to the degree of oral dryness (OR = 15.97; 95% CI: 2.06–123.72). The group with higher scores (OHAT > 8), which represent poor oral conditions, showed severe oral dryness (OR = 17.97; 95% CI: 1.45–223.46). Total OHAT scores (median: 7 vs 2) and those of other subgroups (lip, tongue, gums and tissues, saliva, and oral cleanliness showed a significant decrease after the intervention. Furthermore, the occurrence of mucositis (47.1% vs 0%), candidiasis rate (68.8% vs 43.8%), oral dryness self-sensation (63.6% vs 9.1%), and severe oral debris (52.9% vs 11.8%) decreased significantly.

**Conclusions:**

Proper oral care can improve oral health and hygiene, reduce the rate of mucositis, reduce the sensation of oral dryness, increase oral moisture, and reduce the chances of oral infections among end-of-life patients. Daily oral care is necessary and can alleviate oral discomfort, increase food intake, and increase the chances of communication between end-of-life patients and their families.

**Supplementary Information:**

**Supplementary information** accompanies this paper at 10.1186/s12904-020-00684-0.

## Background

In end-of-life patients with advanced cancers in hospices, oral examination, care, and re-examination after oral care (intervention) are crucial. Hospice care has been introduced and provided in Taiwan for more than 20 years. Interdisciplinary cooperation to meet the needs of hospice patients has received increasing attention. Experts, including psychologists, social workers, nutritionists, pharmacists, and spiritual therapist, work together to provide care to hospice patients. However, as in other countries, regular dental intervention is rare. Although oral care has become routine, and oral hygiene instruction is necessary, insufficient knowledge of oral symptoms, in particular, the inability to diagnosis symptoms early and provide subsequent intervention, causes problems.

In Taiwan, end-of-life oral care still receives less attention from physicians, nurses, caregivers, or patients themselves, similar to other countries [1–4]. In end-of-life patients, probably because of their weakness, oral symptoms are one of their major complaints of discomfort. Symptoms include oral pain, oral candidiasis, angular cheilitis, denture stomatitis, mucositis, dysphagia, ulceration, taste disorders, halitosis, oral infection, and dry mouth, which is the most common oral symptom [3, 5, 6]. The aetiologies of oral problems are numerous, such as general weakness of the patients; previous cancer treatment, including radiotherapy and chemotherapy; present medication; dehydration; and the limited ability of food or water intake; their physical condition may deteriorate because of oral symptoms, which result in dehydration and the inability of food intake [6–8]. Oral care has been reported to be the most effective method of solving oral the problems of patients in hospices [1, 9]. However, studies evaluating the benefits of improvements of oral conditions after oral care are scant.

Most hospice patients in Taiwan have advanced cancers; consequently, the average length of hospice stay of these patients is 10–14 days, which is less than that in other countries. Moreover, at admission to a hospice, these patients are close to the end of their lives. Hence, their survival days are considerably fewer than those of patients in other studies [5, 10]. Considering the aforementioned facts, we can assume that the oral condition of hospice patients in Taiwan is poorer than that of hospice patients in other countries. Therefore, a proper care protocol is necessary for terminally ill patients with advanced cancers. In this prospective study, the associations between oral symptoms and oral dryness among end-of-life hospice patients were explored, and improvements of oral conditions of these patients after an oral care intervention provided by a professional dentist were quantified.

## Methods

### Study design

**Fig. 1:**
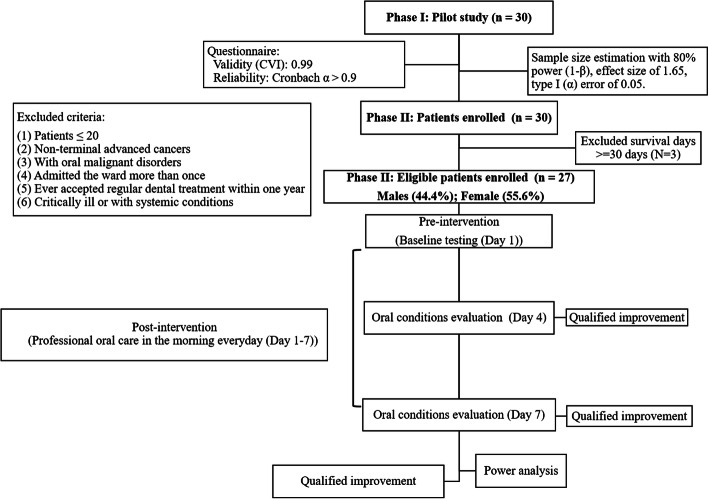
Flow chart of this study

A clinical prospective intervention study in end-of-life patients with advanced cancers was conducted. The study protocol was certified and approved by the Institutional Review Board (IRB)/Ethics Committee at Kaohsiung Medical University (KMU) Chung-Ho Memorial Hospital (IRB Number: KMUHIRB-SV(I)-20,180,050). All patients were admitted in the hospice ward of KMU Hospital between March and August 2019, and informed consent was written by volunteer patients or their primary caregivers. We conducted this study in two phases, and the flow of the study protocol is illustrated in Fig. 1.

### Phase I (pilot study, *n* = 30)

Before providing oral care, we defined inclusion and exclusion criteria, established a method for evaluating the quality of care, confirmed the validity and reliability of our evaluation tool (a questionnaire), and calculated the required sample size. We recruited 30 patients in our Phase I pilot study and confirmed the questionnaire’s validity (0.99) and reliability (Cronbach’s α > 0.9). We also calculated the minimum sample size of 12. Only patients who (1) were ≥ 20 years old, (2) had received a diagnosis of terminal advanced cancers by two specialists, and (3) agreed to accept oral care intervention were included. The patients who (1) were < 20 years old, (2) had non–terminal advanced cancers, (3) had oral malignant disorders, (4) were warded more than once, (5) accepted any regular dental treatment within the past 1 year, and (6) were critically ill or with systemic conditions, such as extremely low platelet count, or those who could not withstand the oral care procedure were excluded.

### Phase II (*n* = 27)

We then began the Phase II study by recruiting 30 patients. However, we excluded three patients who survived for > 30 days and included only end-of-life patients. The exclusion and inclusion criteria and the intervention of oral care procedures were identical to those of the Phase I pilot study. After carefully anticipating and solving possible problems in the pilot study, we collected data on the oral examinations and physical conditions of 27 terminally ill patients with advanced cancers.

Finally, 27 end-of-life patients with advanced cancers were enrolled; they underwent oral examination on Days 1, 4, and 7 in the intervention period. Professional oral care was administered daily in the morning. Before providing oral care, we performed an oral examination. We recorded the oral conditions on Days 1, 4, and 7. Therefore, subsequent oral care would not affect the initial oral examination data. Power analysis was performed, and the improvement of conditions was evaluated by comparing oral conditions after and before the intervention.

### Classification of cancer type

According to International Classification of Diseases for Oncology, Third Edition (ICD-O-3), cancers are coded according to topographic sites. In this study, cancers of various anatomical sites included malignant carcinoma of the colon, rectum, rectosigmoid junction, and anus (C18–C21); the trachea, bronchus, and lungs (C33–C34); the liver and intrahepatic bile ducts (C22); the head and neck (C76); the oesophagus (C15); leukaemia (C42); skin and malignant melanoma (C44); the pancreas (C25); the kidneys (C64); the cervix uteri (C53); the corpus uteri (C54); and the ovary, fallopian tube, and broad ligament (C56, C57.0–C57.4); the female breasts (C50); and the meninges, spinal cord, and other parts of the central nervous system (C70, C72).

### Content and sections of the questionnaire

The final questionnaire content for the patients with cancer contained four sections on their (1a) sociodemographic information (gender, age, and education level) and (1b) clinical index (BMI, estimated survival, Eastern cooperative oncology group (ECOG) status, activities of daily living (ADL), Glasgow coma scale, palliative prognosis index, limb oedema index, and bacterial level), (2) oral health (mucosa health, oral moisture, and oral cleanliness), (3) general oral assessment (Oral Health Assessment Tool (OHAT) at examination at different oral sites), and (4) oral function (voice, swallow, modified water swallowing test (MWST), and maximum mouth opening (MMO)). These sections comprised 19 major items, which are detailed subsequently.

### Oral examination and data recording

All examinations were conducted by a certified dentist. Our oral examination comprised four major sections. The first section involved extracting patient data from medical records. These data comprised general data on cancer type and cancer treatment prior to being warded in the hospice, sociodemographic data (assessed using three questions, each on gender, age, and education level), and clinical index data (BMI, estimated survival, ECOG status, ADL, Glasgow coma scale, palliative prognosis index, limb edema index, and bacterial amount level). Furthermore, data of personal habits including oral care habits, record of ECOG, consciousness status (Glasgow coma scale) [11], and the nutrition route were collected.

The second section was the oral examination, which was used to gather oral health data on mucosa health, oral cleanliness, moisture, and oral function. To evaluate oral mucositis, we used the mucositis classification of World Health Organization (WHO) [12–14]. In this study, we divided the patients into two groups based on the presence or absence of mucositis: the presence of mucositis (grades I–IV) and absence of mucositis (Grade 0). The description of grades is as follows: Grade 0 (no mucositis): no changes or normal mucosa; Grade I (mild mucositis): oral soreness and erythema; Grade II (moderate mucositis): oral erythema, ulcers, and ability to accept a solid diet; Grade III (severe mucositis): oral ulcers and ability to accept a liquid diet only; and Grade IV (life-threatening mucositis): oral alimentation is impossible [13].

To evaluate oral cleanliness, we determined the tongue coating index (TCI) according to a previously reported method [15]. The TCI separated the tongue surface into nine parts, and the consistency of tongue fur or debris on each part was recorded. The level of bacterial accumulation was assessed using a rapid oral bacteria quantification machine through dielectrophoresis and impedance measurement; higher readings represented the higher levels of bacterial accumulation (level 1 indicated an accumulated bacterial count of < 10^5^ CFU/ml^;^ level 2 indicated an accumulated bacterial count between 10^5^ and 10^6^ CFU/ml, level 3 indicated an accumulated bacterial count between 10^6^ and 10^6.5^ CFU/ml, and level 7 indicated an accumulated bacterial count of over 10^8^ CFU/ml) [16]. Therefore, we divided the patients into low (≤10^6.5^ CFU/ml) and high (> 10^6.5^ CFU/ml) groups based on the accumulation of bacteria.

The oral debris, also called the detach mucous film retention in Japan, was classified into three levels according to its amount and consistency. The mild group comprised level 1 (no retention) and level 2 (some retention and white appearance). The severe group comprised level 3 (generalised intraoral retention). Oral moisture was recorded using the Clinical Diagnosis Classification (Grade 0, 1, 2, and 3) of oral dryness and self-reported oral dryness (sensation), according to a previous study [17]. Grade 0 indicated non–dry mouth, Grade 1 viscous saliva in the mouth, Grade 2 saliva with tiny bubbles on the tongue, and Grade 3 dry tongue with either no or less viscous saliva.

Furthermore, oral dryness sensation was assessed by directly asking the patients (scores of 1, 2, and 3 indicated sensations of no, mild, and severe oral dryness) by using a questionnaire. A device was used to measure oral moisture to quantify the degree of oral dryness (Mucus®, Life Co., Ltd.) [18].

To verify the effect of the intervention on oral moisture, we used the oral moisture device before and after the oral care. Oral moisture was tested at two sites, namely 1 cm from the tongue tip on the dorsal tongue and on the buccal mucosa (1 cm from the corner of the mouth). Oral moisture post-test was tested 1–2 h after the intervention had been administered in the morning.

The third section indicated general oral evaluation conducted using the Oral Assessment Guide (OAG) [19] and Oral Health Assessment Tool (OHAT) [20]. The OHAT has eight categories and includes evaluation of the entire mouth; scores of 0, 1, and 2 represent normal oral conditions, change in conditions, and unhealthy conditions, respectively, and the OHAT is the recommended assessment in dental health examinations [20]. Furthermore, the OAG has eight categories of evaluation; however, the evaluation category is partially different and includes differences in description, such as evaluation of voice and swallowing function, and the ratings range from 1 to 3 [19].

The fourth section evaluated voice function, where the patients were divided into normal and abnormal groups; the normal group was assigned a score of 1. The abnormal group had ratings of 2 and 3, respectively, which indicated that the patients had deeper than normal or raspy voices and experienced difficulty in talking or crying or pain while talking or crying. The fourth part includes the swallowing function was assessed by determining the swallowing scores of the OHAT, MWST [21]. The fungal infection examination included collection of tongue smears for *Candida* examination. In most categories, higher scores represented poorer conditions. However, in the MWST, higher scores indicated superior swallowing function. Higher readings on the oral moisture device indicated higher oral moisture.

We considered the first day on which the patients were admitted to the hospice as Day 1. The first oral examination was conducted on Day 1 before any oral care or oral treatment. After the examination, oral care was initiated, and oral hygiene instructions were provided to the patients’ families or caregivers. The oral care procedure we developed combined a wiping technique [22] and oral care protocols suggested by professor Matsuo [10]. Oral examination was performed every 3 days (Days 1, 4, and 7), and professional oral care was provided every morning and three times per day.

### Oral care protocol

At the beginning of oral care, a facial massage was provided, including a massage of the masseter muscle and salivary glands. An oral moisture spray and oral moisture jelly were used for increasing mucosal moisture and for protecting the mucosa. Retained plaque and debris on the teeth were removed using a soft dental brush. Then, oral foam sticks were used for mucosa cleaning. The teeth and mucosa were wiped with a moistened gauze. Oral moisture jelly was applied again for maintaining the moisture of the oral mucosa.

### Data analyses

#### Statistical analysis

All quantitative variables are presented as medium and interquartile range (IQR), and categorical variables are presented as frequencies and percentages. Because these data did not conform to the assumption of normality according to Shapiro–Wilk test, the nonparametric Mann–Whitney *U* was used to test two independent samples. Furthermore, a Friedman test was used to test any significant difference in the measurements over time. For intragroup comparisons, Cochran’s Q for repeated measures was used to assess bivariate associations. After controlling for gender and age, adjusted odds ratio (AOR), 95% confidence intervals (CI) and exact *p* values were estimated with an unconditional logistic regression model. To confirm agreement between the examiner’s and senior dentist’ evaluations, Cohen’s kappa statistics (an interrater statistical indicator) and overall percentage agreement were calculated. In this study, *two*-*tailed p* < 0.05 was considered statistically significant. All statistical procedures were conducted using SPSS v 20.0 Statistical Package (SPSS Inc., IBM, Armonk, New York, USA).

#### Sample size calculation and the power of the study

G∗Power (version 3.1.9.4, program written, conceptualised, and designed by Franz, Universitat Kiel, Germany), a freely available windows application software, was used for sample size and power estimation. Our pilot study estimated an effect size of 1.65; the mean ± standard deviation for SOD was 1.87 ± 0.35 and that for nonsevere oral dryness (NOD) was 1.07 ± 0.59. In our pilot study’s nonparametric Mann–Whitney *U* test, the estimated effect size was 1.65, type I (α) error was 0.05, and type II error (β) was 0.2; a minimum sample size of 12 was required to achieve a power (1-β) of ≥80%. Thus, in total, 27 participants were recruited to this study, and post hoc power analyses indicated that post hoc power was at least 85%, and a type II error (β) of 0.15 was present.

## Results

### Validity and reliability of the questionnaire

In our pilot study, a multidisciplinary expert panel evaluated the face and content validity index (CVI) of the questionnaire. A total of 30 completed questionnaires were collected in our pilot study. Question items with a validity score of > 3 were retained in the final questionnaire, and CVI was calculated for the items in each scale. The validity of all questionnaire items was evaluated by six specialists, and a high CVI was obtained (CVI = 0.99). Questionnaire reliability was assessed using internal consistency reliability. Internal consistency was estimated using Cronbach α, and a Cronbach α of more than 0.8 was acceptable. In our questionnaire, internal consistency reliability was high (Cronbach α > 0.9).

### Calibration of professional dentists

**Fig. 2:**
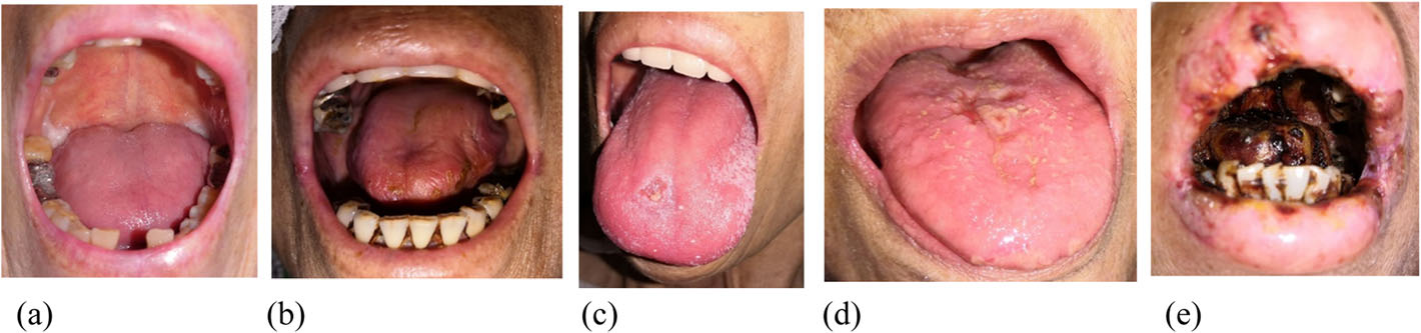
The mucositis classification of WHO was examined by professional dentist. The presence of mucositis (grades I–IV) and absence of mucositis (grade 0) in end-of-life patients with advanced cancers. Representative diagrams: (**a**) No mucositis, (**b**) WHO grade 1, (**c**) WHO grade 2, (**d**) WHO grade 3; (**e**) WHO grade 4

**Fig. 3:**
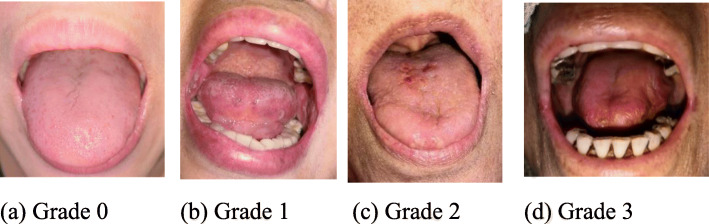
The Clinical Diagnosis Classification of oral dryness patients was examined by professional dentist in end-of-life patients with advanced cancers. Representative diagrams: (**a**) Grade 0, (**b**) Grade 1, (**c**) Grade 2; (**d**) Grade 3

The examiner had excellent agreement with the senior dentist in the reference standard judgement method. Between the two dentists, their perfect agreement kappa scores were 0.945 for the consistency between their diagnoses of the WHO mucositis classification (*p* < 0.001) and 0.847 for that of the Clinical Diagnosis Classification of oral dryness (*p* < 0.001). The representative diagrams of the oral conditions of mucositis and oral dryness are presented in Figs. 2 and 3.

### Study patient characteristics

Table 1 shows the distribution of clinical characteristics and cancer type between the sexes. Finally, a total of 27 end-of-life patients (men = 12 [44.4%]; women = 15 [55.6%]) from in the hospice ward of KMU Hospital were included. All our patients were diagnosed with advanced cancers and had a survival of <=30 days (minimum = 2; maximum = 23). The median of age was 61 and 68 years among the men and women, respectively. The median of estimated survival times was 9 and 12 days for the men and women, respectively. The results revealed that the men and women did not differ significantly in terms of clinical and demographic characteristics (such as age, education level, BMI, survival time, ECOG status, ADL scores, Glasgow coma scores, palliative prognosis index, limb oedema index, bacterial count, and cancer type distribution).

**Table 1:** The clinical characteristics and cancer type distribution between males and females in end-of-life patients with advanced cancers

Characteristics	End-of-life patients (*n* = 27)						χ^2^ / Fisher’s exact test	Mann- Whitney *U*
	Total		Males (*n =* 12)		Females (*n =* 15)			
	n (%)/ Median (IQR)						*p*	*p*^b^
**Clinical index**								
Age (yrs)	62.0	(58.0–72.0)	61.0	(58.0–72.8)	68.0	(60.0–72.0)		0.462
Education level (yrs)								
≤ 9	13	(59.1%)	7	(70.0%)	6	(50.0%)	0.415^a^	
> 9	9	(40.9%)	3	(30.0%)	6	(50.0%)		
BMI	21.0	(20.0–25.0)	22.0	(20.0–25.3)	21.0	(17.0–25.0)	1.000	
Estimated survival (days)	9.0	(4.0–19.0)	9.0	(2.5–18.5)	12.0	(4.0–19.0)		0.509
ECOG status (bedridden time(%))								
< 100%	12	(44.4%)	5	(41.7%)	7	(46.7%)	0.795^a^	
100%	15	(55.6%)	7	(58.3%)	8	(53.3%)		
ADL	0.0	(0.0–10.0)	0.0	(0.0–0.0)	0.0	(0.0–15.0)		0.290
Glasgow coma scale	14.0	(9.0–15.0)	14.5	(5.0–15.0)	14.0	(12.0–15.0)		0.701
Palliative prognosis index	8.0	(6.0–14.3)	10.5	(5.0–14.8)	8.0	(6.0–11.3)		0.547
Limb edema index (0–16 scores)	3.0	(0.5–7.0)	2.0	(0.0–4.0)	5.0	(1.0–7.0)		0.154
Limb edema								
Index > 0	5	(19.2%)	3	(27.3%)	2	(13.3%)	0.620^a^	
Index =0	21	(80.8%)	8	(72.7%)	13	(86.7%)		
Bacterial amount level								
Low	17	(65.4%)	10	(83.3%)	7	(50.0%)	0.110^a^	
High	9	(34.6%)	2	(16.7%)	7	(50.0%)		
**Cancer type distribution**								
Colon, rectum, rectosigmoid junction and anus (%)	7	(25.9%)	2	(16.7%)	5	(33.3%)	0.612^a^	
Trachea, bronchus and lung (%)	6	(22.2%)	2	(16.7%)	4	(26.7%)		
Liver and intrahepatic bile ducts (%)	3	(11.1%)	2	(16.7%)	1	(6.7%)		
Others	11	(40.7%)	6	(50.0%)	5	(33.3%)		

Data are n (%) or median (IQR)

Abbreviations: *IQR* interquartile range, *BMI* Body Mass Index, *ECOG* Eastern Cooperative Oncology Group, *ADL* Activities of Daily Living

^a^Fisher’s exact test

^b^Asymptotic significance by Mann- Whitney *U* test

In total, 25.9% of the participants were diagnosed with cancers of the colon, rectum, rectosigmoid junction, and anus; 22.2% with cancers of the trachea, bronchus, and lungs; 11.1% with cancers of the liver and intrahepatic bile ducts; and 40.7% with other cancers. Among the men, the distribution of cancers was as follows: 16.7% of the patients had cancers of the colon, rectum, rectosigmoid junction, and anus; 16.7% had cancers of the trachea, bronchus, and lung; 16.7% had cancers of the liver and intrahepatic bile ducts; and 50.0% had other cancers, which included cancers of the esophagus and pancreas, multiple myeloma, and skin cancer and malignant melanomas. The distribution of cancers among the women was as follows: 33% of the patients had cancers of the colon, rectum, rectosigmoid junction, and anus; 26.7% had cancers of the trachea, bronchus, and lungs; 6.7% had cancers of the liver and intrahepatic bile ducts; and 33.3% had other cancers, which included those of the female genital organs; female breasts; and meninges, spinal cord, and other parts of the central nervous system. Female genital organs included the cervix uteri, corpus uteri, and ovaries, fallopian tubes, and broad ligament.

Table 2 presents a comparison of the distribution of demographic characteristics and oral symptoms between the patients with NOD and SOD. No significant differences were observed in the distributions of sex, age, education levels, BMI, and survival days between the two groups. Regarding oral conditions, significant differences were observed in mucositis, oral dryness, self-perceived oral dryness, oral debris retention level, some OHAT scores, and voice function. In the SOD group, 66.7% of the patients exhibited Grade I mucositis, which was significantly higher than the proportion of the patients in the NOD group (*p* = 0.007). All patients with SOD complained of a feeling of oral dryness. Accordingly, the sensation of oral dryness was also significantly more severe in the SOD group than in the NOD group (*p* = 0.007). The oral moisture readings of the tongue and buccal mucosa were significantly higher in the NOD group (*p* < 0.05) than in the SOD group. The readings of the tongue showed higher significant differences than those of the buccal mucosa (*p* = 0.002 vs *p* = 0.014) between the two groups. A higher prevalence (75%) of a high level of retention of oral debris was observed in the SOD group than in the NOD group (*p* = 0.004). With regard to oral function, only voice function disturbances were significantly higher in the SOD group than in the NOD group (*p* = 0.047).

**Table 2:** Distribution of clinical indexes and oral conditions between non-severe and severe oral dryness in end-of-life patients with advanced cancers

	End-of-life patients (*n* = 27)				χ^2^ / Fisher’s exact test	Mann- Whitney *U*
	Oral dryness					
	NOD (*n =* 15)		SOD (*n =* 12)			
	n (%)/ Median (IQR)				*p*	*p*^b^
**Clinical index**						
Gender						
Males	7	(46.7%)	5	(41.7%)	0.795	
Females	8	(53.3%)	7	(58.3%)		
Age (yrs)	62.0	(58.0–70.0)	67.0	(58.5–72.8)		0.806
Education level (yrs)						
≤ 9	7	(53.8%)	6	(66.7%)	0.674^a^	
> 9	6	(46.2%)	3	(33.3%)		
BMI	21.0	(20.0–23.0)	24.0	(17.8–26.8)		0.247
Estimated survival (days)	9.0	(4.0–21.0)	10.0	(4.3–15.0)		0.826
ECOG status (bedridden time(%))						
< 100%	6	(40.0%)	6	(50.0%)	0.603	
100%	9	(60.0%)	6	(50.0%)		
ADL	0.0	(0.0–30.0)	0.0	(0.0–0.0)		0.139
Glasgow coma scale	14.0	(8.0–15.0)	14.0	(9.3–15.0)		0.878
Palliative prognosis index	8.0	(4.0–12.8)	10.0	(8.0–14.8)		0.209
Limb edema index (0–16 scores)	2.0	(0.0–4.0)	6.0	(2.3–7.8)		0.100
Limb edema						
Index > 0	4	(26.7%)	1	(9.1%)	0.356^a^	
Index =0	11	(73.3%)	10	(90.9%)		
Bacterial amount level						
Low	8	(53.3%)	9	(81.8%)	0.217^a^	
High	7	(46.7%)	2	(18.2%)		
**Oral conditions**						
Mucosa healthiness						
Mucositis						
(−)	13	(86.7%)	4	(33.3%)	0.007^a^*	
(+)	2	(13.3%)	8	(66.7%)		
Candida infection						
(+)	9	(60.0%)	10	(83.3%)	0.236^a^	
(−)	6	(40.0%)	2	(16.7%)		
Oral bleeding						
(+)	3	(20.0%)	1	(8.3%)	0.61^a^	
(−)	12	(80.0%)	11	(91.7%)		
Oral moisture						
Sensation of oral dryness						
(+)	5	(41.7%)	9	(100.0%)	0.007^a^*	
(−)	7	(58.3%)	0	(0.0%)		
Tongue with oral moisture machine at initial examination	27.1	(22.1–29.2)	0.0	(0.0–5.6)		0.002*
Buccal mucosa with oral moisture machine at initial examination	28.5	(26.1–31.0)	20.4	(0.0–27.5)		0.014*
Oral cleanliness						
Oral debris						
Mild	12	(80.0%)	3	(25.0%)	0.004*	
Severe	3	(20.0%)	9	(75.0%)		
Tongue coating index	0.4	(0.3–0.6)	0.4	(0.3–0.6)		1.000
**General oral assessment**						
OHAT at initial examination						
Total scores	6.0	(5.0–8.0)	8.5	(6.3–9.8)		0.014*
Lips	1.0	(1.0–2.0)	1.0	(1.0–2.0)		0.422
Tongue	1.0	(1.0–1.0)	1.0	(1.0–2.0)		0.019*
Gums and tissues	0.0	(0.0–1.0)	1.0	(1.0–1.0)		0.011*
Saliva	1.0	(1.0–1.0)	2.0	(2.0–2.0)		< 0.001*
Natural teeth	1.0	(0.0–2.0)	1.0	(0.0–1.0)		0.437
Dentures	0.0	(0.0-NA)	1.5	(0.3–2.0)		0.190
Oral cleanliness	1.0	(1.0–2.0)	1.5	(1.0–2.0)		0.476
Dental pain	0.0	(0.0–0.0)	0.0	(0.0–0.0)		0.371
**Oral function**						
Voice						
Normal	5	(33.3%)	0	(0.0%)	0.047^a^*	
Abnormal	10	(66.7%)	12	(100.0%)		
Swallow						
(+)	8	(57.1%)	8	(66.7%)	0.701^a^	
(−)	6	(42.9%)	4	(33.3%)		
MWST						
Swallowing with neither cough nor wet hoarseness	4	(57.1%)	2	(40.0%)	1.00^a^	
In addition to 4 points, could swallow saliva two additional times within the 30 s	3	(42.9%)	3	(60.0%)		
MMO	3.5	(3.0–4.0)	3.0	(2.0–3.5)		0.180

Data are n (%) or median (IQR). *NA* not applicable

Abbreviations: *IQR* interquartile range, *NOD* Nonsevere Oral Dryness, *SOD* Severe Oral Dryness, *BMI* Body Mass Index, *ECOG* Eastern Cooperative Oncology Group, *ADL* Activities of Daily Living, *OHAT* Oral Health Assessment Tool, *MWST* Modified Water Swallowing Test, *MMO* Maximum Mouth Opening

^a^Fisher’s exact test

^b^Asymptotic significance by Mann- Whitney *U* test

* Statically significant (*p* < 0.05)

To understand the associations between oral condition factors and the degree of oral dryness, we used logistic regression adjusted for the age and sex covariates (Table 3). The presence of oral mucositis was strongly associated with SOD. The patients with oral mucositis had a higher probability of SOD (AOR = 14.93; 95% CI: 1.95–114.38; *p* = 0.009) than did those without. We noted that the level of oral debris was significantly related to the degree of oral dryness (AOR = 15.97; 95% CI: 2.06–123.72; *p* = 0.008). Because of the total score of the OHAT was 16 points, we divided the patients into two groups according to their total OHAT scores with a cutoff point of 8 (16 divided by 2). Scores of ≤8 indicated healthy oral conditions, and scores of > 8 indicated unhealthy oral conditions. The result showed that high OHAT scores (OHAT > 8) were significantly associated with SOD (AOR = 17.97; 95% CI: 1.45–223.46; *p* = 0.008).

**Table 3:** Oral conditions associated with non-severe and severe oral dryness in end-of-life patients with advanced cancers

	End-of-life patients (*n =* 27)							
	Oral dryness							
	NOD (*n =* 15)		SOD (*n =* 12)					
	n	(%)	n	(%)	OR^a^	95% CI	AOR^b^	95% CI
Mucositis								
(−)	13	(86.7%)	4	(33.3%)	1.00		1.00	
(+)	2	(13.3%)	8	(66.7%)	13.00	(1.92–87.99)*	14.93	(1.95–114.38)*
OHAT total score								
< =8	14	(93.3%)	6	(50.0%)	1.00		1.00	
> 8	1	(6.7%)	6	(50.0%)	14.00	(1.37–142.89)*	17.97	(1.45–223.46)*
Oral debris								
Mild	12	(80.0%)	3	(25.0%)	1.00		1.00	
Severe	3	(20.0%)	9	(75.0%)	12.00	(1.95–73.97)*	15.97	(2.06–123.72)*

Data are n (%)

Abbreviations: *NOD* Nonsevere Oral Dryness, *SOD* Severe Oral Dryness, *OR* Odds Ratio, *AOR* Adjusted Odds Ratio, 95% *CI* 95% confidence interval

^a^Odds ratios (OR) refer to risk of severe oral dryness versus non-severe oral dryness patients according to each variable by logistic regression model with OR, and 95% CI. OR > 1 indicates a higher likelihood of being a severe oral dryness

^b^AOR: adjusted odds ratio for gender, and age (years) by logistic regression model with AOR, and 95% CI. AOR > 1 indicates a higher likelihood of being a severe oral dryness

As shown in Table 4, the improvement in oral symptom scores was demonstrated by the comparison of the initial scores (Day 1) with those after oral care interventions on Days 4 and 7; the scores were recorded every 3 days before routine oral care. Significant improvement of oral conditions was observed with regard to mucositis, *Candida* infection, sensation of oral dryness, and oral debris retention levels. Cochran’s Q test was used to evaluate the proportion change in repeated measures; the mucositis rate, which was 47.1% on Day 1, significantly decreased to 0% on Day 7 (*p* = 0.003). The candidiasis rate significantly decreased from 68.8% before the intervention to 43.8% after the intervention (*p* = 0.015). The proportion of sensation of oral dryness decreased from Day 1 (63.6%) to Day 7 (9.1%). Furthermore, the level of oral debris retention significantly decreased from 52.9 to 11.8% (*p* = 0.001). The median of the TCI decreased from 0.3 to 0.2, which was a marginally significant decrease (*p* = 0.065). In general, in the oral assessment, significant differences were observed in the total OHAT scores and the scores in the categories of lips, tongue, gums and tissue, saliva, and oral cleanliness. The median of total OHAT score was 7 on Day 1, and it significantly decreased to 2 on Day 7 (*p* < 0.001). No significant improvement was observed in the outcomes of oral function examination.

**Table 4:** The improvement of oral symptom scores before and after oral care interventions in end-of-life patients with advanced cancers

	End-of-life patients (*n =* 27)							
	Oral care interventions							
	Pre-intervention		Post-intervention					
	Day 1		Day 4	Day 7		Cochran’s Q test	Friedman test	
	n (%)/ Median (IQR)					*p*	*p*	
**Oral conditions**								
Mucosa healthiness								
Mucositis								
(+)		47.1%^a^		11.8%^b^		0.0%^b^	0.003*	
(−)		52.9%		88.2%		100.0%		
Candida infection								
(+)		68.8%^a^		37.5%^b^		43.8%	0.015*	
(−)		31.3%		62.5%		56.3%		
Oral bleeding								
(+)		17.6%		5.9%		0.0%	0.097	
(−)		82.4%		94.1%		100.0%		
Oral moisture								
Oral dryness								
SOD		41.2%		35.3%		29.4%	0.741	
NOD		58.8%		64.7%		70.6%		
Sensation of oral dryness								
(+)		63.6%^a^		27.3%		9.1%^b^	0.009*	
(−)		36.4%		72.7%		90.9%		
Oral cleanliness								
Oral debris								
Severe		52.9%^a^		5.9%^b^		11.8%^b^	0.001*	
Mild		47.1%		94.1%		88.2%		
Tongue coating index	0.3	(0.3–0.7)	0.3	(0.1–0.3)	0.2	(0.1–0.5)		0.065
**General oral assessment**								
OHAT at initial examination								
Total scores	7.0	(6.0–9.0)^a^	3.0	(1.0–4.0)^b^	2.0	(0.0–4.0)^b^		< 0.001*
Lips	1.0	(1.0–2.0)^a^	0.0	(0.0–0.5)^b^	0.0	(0.0–0.0)^b^		< 0.001*
Tongue	1.0	(1.0–1.5)^a^	1.0	(0.0–1.0)	1.0	(0.0–1.0)^b^		0.003*
Gums and tissues	2.0	(1.5–2.0)^a^	1.0	(1.0–1.0)^b^	1.0	(1.0–1.0)^b^		< 0.001*
Saliva	1.0	(1.0–2.0)^a^	1.0	(0.0–1.0)	1.0	(0.0–1.0)^b^		0.001*
Natural teeth	1.0	(0.0–2.0)	1.0	(0.0–2.0)	1.0	(0.0–2.0)		1.000
Dentures	0.0	(0.0–2.0)	0.0	(0.0–1.5)	0.0	(0.0–1.5)		0.368
Oral cleanliness	1.0	(1.0–2.0)^a^	1.0	(0.0–1.0)	1.0	(0.0–1.0)^b^		0.002*
Dental pain	0.0	(0.0–0.0)	0.0	(0.0–0.0)	0.0	(0.0–0.0)		0.368
**Oral function**								
Voice								
Abnormal		82.4%		76.5%		70.6%	0.549	
Normal		17.6%		23.5%		29.4%		
Swallow								
(+)		62.5%		81.3%		81.3%	0.165	
(−)		37.5%		18.8%		18.8%		
MMO	3.5	(2.5–4.0)	3.0	(2.0–3.8)	3.0	(3.0–3.5)		0.731

Data are (%) or median (IQR)

Abbreviations: *IQR* interquartile range, *NOD* Nonsevere Oral Dryness, *SOD* Severe Oral Dryness, *OHAT* Oral Health Assessment Tool, *MMO* Maximum Mouth Opening

^a, b^Different upper case letters denote significant differences (*p* < 0.05) between groups by Cochran’s Q (asymptotic significances) / Friedman test (asymptotic significances) and their correspondent post hoc comparisons

## Discussion

Interdisciplinary cooperation has become a trend in hospice care. Increasing attention is being paid to oral care, rehabilitation, or treatments based on traditional Chinese Medicine for terminally patients. Oral symptoms are among the major complaints of hospice patients. Most patients in hospices have advanced cancers and are at the end of their lives; they have experienced a long and difficult fight against cancer. Because of the general weakness and pain experienced by patients, side effects of cancer treatment or surgery, or depression, most patients ignore their oral health. Consequently, most patients have been reported to show poor oral health at admission into hospice wards [2, 3, 5, 6, 10, 23–25]. Among our patients, only 2 patients accepted a routine dental follow-up during the past 1–2 years. Although we excluded patients who received regular oral care and examinations, we did not encounter any patients who continued to undergo oral examinations and care after the last diagnosis of cancer.

During our procedure of enrolment, SOD, *Candida* infections, generalised oral ulcers, mucositis, lack of proper products for administering care [1], and uncooperative behaviour of patients during oral care administration, which was mainly because of their unconsciousness, were the most common problems faced by the care team; hence, providing continued the oral care is difficult. Despite the multitude of difficulties, administering oral care to terminally ill patients is necessary. In a previous study, oral care was shown to increase oral moisture levels, provide relief from oral symptoms, provide a sensation of oral comfort, reduce the degree of dysgeusia, reduce the chances of odontogenic infections, increase the amount of food intake, and improve quality of life [4, 5, 8, 24–26]. Some conclusions were also provided by this present study. The median (IQR) of survival days of our patients was 9 days (4–19), and the mean (SD) was 10.85 (7.44). The average survival times were shorter than those in other countries [5, 10]. The difference in survival times is mainly because of the law of public health insurance in Taiwan. According to the previous study, patients with fewer residual days have more severe oral symptoms, such as tongue inflammation, dry mouth, and oral bleeding spots, than other patients [10]. Consequently, we hypothesised that the general and oral conditions of our patients at the beginning of the study were poor.

In Table 2, oral dryness severity did not show a significant relationship with demographic characteristics, and the survival days did not differ significantly between the NOD and SOD groups. The oral mucositis rate was significantly higher in the SOD group, and the patients in the SOD group experienced oral dryness. The moisture device readings were similar to the results of the clinical diagnosis in terms of the degree of oral dryness. Although the significance of moisture readings on the tongue was higher, both tongue and buccal mucosa readings were significant. We believe that the use of the moisture device is a reliable method for testing the degree of oral moisture in end-of-life patients. When we could not record readings for the tongue, the readings of the buccal mucosa were satisfactory substitutes.

Notably, the rate of severe oral debris retention was higher in the SOD than in the NOD group, and after adjusting for sex and age, we also found a significant association between severe oral debris retention and SOD (AOR = 15.97). Thus, the retention of debris might be a reliable index of oral dryness. Indeed, a previous study indicated that the score evaluation of clinical oral dryness can include the item of debris on palate (excluding under denture) [27]. The presence of oral mucositis was closely associated with severe oral dryness; the patients with oral mucositis had a higher chance of exhibiting SOD (AOR = 14.93). One of the signs of mucositis is oral dryness, and patients with cancer who have undergone treatment (chemotherapy and/or radiotherapy) often experience simultaneous oral dryness and mucositis [8, 28]. Particularly, the results showed that the high-score (> 8) group, with unhealthy oral conditions, showed a significant association with SOD (AOR = 17.97).

In addition, the voice function was abnormal in the SOD group. The patients with SOD all experienced disturbances while talking; however, no difference was observed in swallowing function between the SOD and NOD groups. After oral care interventions, significant improvements were observed between Days 1 and 7 in the total OHAT scores and the scores in the categories of lips, tongue, gums and tissue, saliva, and oral cleanliness. The WHO mucositis grade, percentage of candidiasis, self-sensation of oral dryness, and oral debris retention rate showed significant improvements. Although Clinical Diagnosis Classification of oral dryness did not significantly change after oral care interventions, a decrease in oral dryness was observed (from 41.2 to 29.4%).

The general physical condition might not improve during the last few days of survival of end-of-life patients. However, the general oral condition, evaluated using OHAT scores, improved in terms of increase in the level of oral cleanliness, decrease in mucositis, and decrease in the oral infection rate after oral care interventions. After oral care interventions, the degree of oral dryness and readings of the moisture device, and index of voice did not differ significantly, but a trend of improvement was observed, and improvement increased with the number of intervention days. The MMO and swallowing function did not show significant improvement, which is reasonable, because of the continuous deterioration of the patients’ physical condition. Some researchers have stated that the oral dryness and dysphagia of the advanced cancer patients were two symptoms that did not improve [29, 30]. In the future, if suitably designed techniques and products for oral care, such as an appropriate moisturising agent, are used, treatment will ameliorate oral dryness and dysphagia. During the enrolment of patients, we noticed that the oral dryness might not be actually improved, but the symptoms accompanying oral dryness and the feeling of oral dryness can be relieved through oral care. With regard to dysphagia, generalised palliative care, posture adjustment of patients, and assisting treatments (massage) might help to maintain the swallowing function for a longer period before the patients really enter the pre-dying stage.

### Strengths and limitations

The strengths of the present study are that it is a prospective intervention study and that we could design an oral care protocol applicable to end-of-life patients with advanced cancers. Careful daily oral examination and re-examination in end-of-life patients are necessary. To the best of our knowledge, the present study is the first to describe intensive oral care interventions in end-of-life patients whose median of survival time was shorter than 30 days as well as the use of a bacteria counter and device to assess oral moisture in palliative oral care. The amount of moisture and bacterial count in the mouth were quantified simultaneously, and each patient received standardised professional oral care once every morning. Each patient was examined and accepted oral care by a professional dentist once a day in the morning. After the intervention period, oral care was provided by their caregivers, who were trained by the same dentist. The caregivers received oral hygiene instructions from the dentist, and the procedure and products used by the caregivers were the same as those used by the dentist. The diagnosis of candidiasis was conducted using a smear test instead of using visible signs. Furthermore, an oral moisture device and bacterial counter were used in the oral examinations for quantifying the improvement of the oral conditions.

The limitation of our study is similar to that of other studies on oral care in hospice patients; the sample size was small and patients dropped out because they were terminally ill. The level of consciousness of our patients also affected the oral examination, 10 of our patients were unable to accept the oral moisture test on the tongue because they were unconscious, but the buccal mucosa provided reliable substitute data. Although our sample size was only 27, the power estimation of this study was > 85%, which indicated an 85% valid probability of significant results. This high statistical power indicated that our test results are likely valid.

**Fig. 4:**
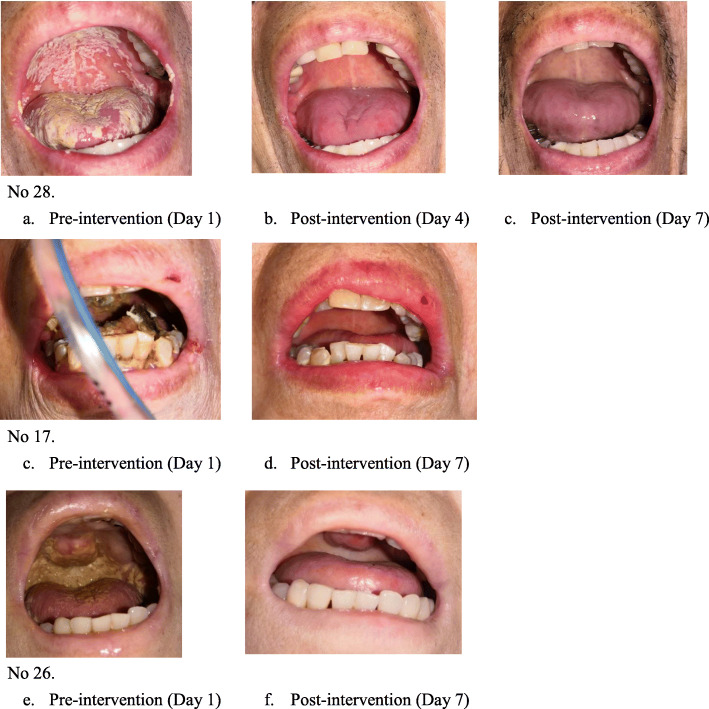
The improvement effect of oral care in representative unconscious patients (*n =* 3). Representative diagrams: In No. 28 patient, (**a**). Pre-intervention (Day 1) (**b**). Post-intervention (Day 4) (**c**). Post-intervention. In No. 17 patient, (**c**). Pre-intervention (Day 1) (**d**). Post-intervention (Day 7). In No. 26 patient, (**e**). Pre-intervention (Day 1) (**f**). Post-intervention (Day 7)

For unconscious patients (*n* = 10) in the beginning, we requested informed consent from their families after thoroughly explaining the study to their families; only then did we perform the procedure. This allowed us to evaluate these patients’ oral conditions (mucosa healthiness, oral moisture, and oral cleanliness) and undertake a general oral assessment (OHAT at initial examination in different oral sites). Only patients’ oral function, such as voice, swallow, and MWSTs could not be checked by the certified dentist. Furthermore, in 10 unconscious patients, their improvements in oral symptom scores were assayed, and we compared their initial scores (Day 1) with those at Days 4 and 7 after the oral care interventions; the scores were recorded every 3 days before routine oral care (Supplement Table 1). Significant improvement was observed in the oral conditions of mucositis, and oral debris retention levels. In particular, the mucositis rate significantly decreased from 57.1% on Day 1 to 0% on Day 7 (*p* = 0.018). Furthermore, the level of oral debris retention significantly decreased from 85.7 to 28.6% (*p* = 0.015). In general, in the oral assessment, significant differences were observed in the total OHAT scores. The median total OHAT score was 8 on Day 1, and it significantly decreased to 3.5 on Day 7 (*p* < 0.001). The qualitative data of improvement effect of oral care in three representative unconscious patients (*n* = 3) were shown in Fig. 4.

In conscious patients, during the care process, we used the assessment of care comfort for conscious patients, per a previous study [25]. Of the 17 conscious patients, 15 felt comfortable or slightly comfortable. Only one patient felt mild discomfort during the oral care procedure. At 3 days after the oral care procedure, 10 patients experienced a decrease in their feeling of dry mouth, indicating that our oral care intervention treated dry mouth and improved patient comfort in their mouth.

Although the oral care protocol was performed three times daily, the oral care protocol was short at approximately 5–10 min each time. Although having to execute the protocol entails greater labour on the part of caregivers, this short duration makes such labour manageable. Family members and caregivers noted the following two points in their feedback. First, the largest psychological burden on them was their unfamiliarity with oral care. However, after our oral hygiene instruction, oral care became much easier for them. Second, family members and caregivers reported feeling unable to help the patient. However, the oral care yielded good results in a short time, which alleviated such concerns. Thus, the increased workload from three times daily oral care is more offset by the reduced psychological burden and increased sense of participation in patient care, making our oral care procedure worth undertaking for family members and caregivers.

## Conclusions

In end-of-life patients with advanced cancers, the presence of oral mucositis, severe oral debris retention, and poor oral conditions (OHAT scores of > 8) were closely associated with SOD. The interventions of professional oral care can reduce the level of retention of oral debris, self-sensation of oral dryness, rate of mucositis, and rate of the candidiasis as well as improve oral assessment OHAT scores and increase oral moisture. Oral care interventions are crucial for patients because they efficiently reduce oral discomfort in the patients, increasing their ability of food intake and increasing their chances of communicating with their families at the end of life.

## Supplementary Information

**Additional file 1: Supplementary Table 1** The improvement of oral symptom scores before and after oral care interventions in unconscious patients.

## Data Availability

The data of this study was used and/or analysed are available from our corresponding author (Shun-Te Huang and Ping-Ho Chen) for a reasonable request.

## Abbreviations

IRB: Institutional Review Board
ICD-O-3: International Classification of Diseases for Oncology, Third Edition
ECOG: Eastern Cooperative Oncology Group
OAG: Oral Assessment Guide
OHAT: Oral Health Assessment Tool
WHO: World Health Organization
TCI: Tongue Coating Index
SOD: Severe Oral Dryness
MWST: Modified Water Swallowing Test
CVI: Content Validity Index
IQR: Interquartile Range
AOR: Adjusted odds ratio
CI: Confidence Intervals
ADL: Activities of Daily Living
NOD: Nonsevere Oral Dryness
MMO: Maximum Mouth Opening
