# Supplementary material for: Professional oral care in end-of-life patients with advanced cancers in a hospice ward: improvement of oral conditions
Source: BMC Palliat Care. 2020 Nov 27;19:181. doi: 10.1186/s12904-020-00684-0 (PMC7697385; doi:10.1186/s12904-020-00684-0)
Supplement: Supplementary file 1 — Additional file 1: Supplementary Table 1 The improvement of oral symptom scores before and after oral care interventions in unconscious patients. [file 12904_2020_684_MOESM1_ESM.docx]

**Supplementary Table 1** The improvement of oral symptom scores before and after oral care interventions in unconscious patients

|  | Unconscious patients (n = 10) | | | | | |  |  |
| --- | --- | --- | --- | --- | --- | --- | --- | --- |
|  | Oral care interventions | | | | | |  |  |
|  | Pre-intervention | |  | Post-  intervention | | |  |  |
|  |  | Day 1 |  | Day 4 |  | Day 7 | Cochran’s Q test | Friedman test |
|  | n (%)/ Median (IQR) | | | | | | *p* | *p* |
| **Oral conditions** |  |  |  |  |  |  |  |  |
| Mucosa healthiness |  |  |  |  |  |  |  |  |
| Mucositis |  |  |  |  |  |  |  |  |
| (+) |  | 57.1%^a^ |  | 0.0%^b^ |  | 0.0%^b^ | 0.018* |  |
| (–) |  | 42.9% |  | 100.0% |  | 100.0% |  |  |
| Candida infection |  |  |  |  |  |  |  |  |
| (+) |  | 66.7% |  | 50.0% |  | 50.0% | 0.368 |  |
| (–) |  | 33.3% |  | 50.0% |  | 50.0% |  |  |
| Oral bleeding |  |  |  |  |  |  |  |  |
| (+) |  | 42.9% |  | 14.3% |  | 0.0% | 0.097 |  |
| (–) |  | 57.1% |  | 85.7% |  | 100.0% |  |  |
| Oral moisture |  |  |  |  |  |  |  |  |
| Oral dryness |  |  |  |  |  |  |  |  |
| SOD |  | 42.9% |  | 42.9% |  | 28.6% | 0.779 |  |
| NOD |  | 57.1% |  | 57.1% |  | 71.4% |  |  |
| Oral cleanliness |  |  |  |  |  |  |  |  |
| Oral debris |  |  |  |  |  |  |  |  |
| Severe |  | 85.7%^a^ |  | 14.3%^b^ |  | 28.6% | 0.015* |  |
| Mild |  | 14.3% |  | 85.7% |  | 71.4% |  |  |
| Tongue coating index | 0.5 | (0.3-0.8) | 0.2 | (0.1-0.3) | 0.4 | (0.1-0.4) |  | 0.102 |
| **General oral assessment** |  |  |  |  |  |  |  |  |
| OHAT at initial examination |  |  |  |  |  |  |  |  |
| Total scores | 8.0 | (5.8-9.0)^a^ | 3.5 | (1.5-5.0)^b^ | 3.5 | (0.0-4.0)^b^ |  | <0.001* |
| Note: Data are (%) or median (IQR).  *Abbreviations: IQR* interquartile range, *NOD* Nonsevere Oral Dryness, *SOD* Severe Oral Dryness, *OHAT* Oral Health Assessment Tool, *MMO* Maximum Mouth Opening  ^a,b^ Different upper case letters denote significant differences (*p*<0.05) between groups by Cochran’s Q (asymptotic significances) / Friedman test (asymptotic significances) and their correspondent post hoc comparisons | | | | | | | | |
